# Higher Prevalence of Diabetes in Pontine Infarction than in Other Posterior Circulation Strokes

**DOI:** 10.1155/2022/4819412

**Published:** 2022-01-27

**Authors:** Jinmao Zhu, Youfu Li, Yanxia Wang, Shuanggen Zhu, Yongjun Jiang

**Affiliations:** ^1^Department of Neurology, The Second Affiliated Hospital of Guangzhou Medical University, 250 Changgang East Road, Guangzhou 510260, China; ^2^Department of Neurology, People's Hospital of Longhua, Shenzhen 518109, China

## Abstract

**Background:**

Pontine infarction is the major subtype of posterior circulation stroke, and diabetes is more common in pontine infarction patients than in anterior circulation stroke patients. Whether the prevalence of diabetes remains homogenous within the posterior circulation stroke population is unclear. The present study is aimed at investigating the prevalence of diabetes in pontine infarction and comparing it to other subtypes of posterior circulation stroke.

**Methods:**

We conducted a multicenter case-control study. Patients with posterior circulation stroke were screened. The subjects were divided into pontine infarction and nonpontine infarction groups.

**Results:**

From November 1, 2018, to February 28, 2021, a total of 6145 stroke patients were screened and 2627 patients had posterior circulation strokes. After excluding cardioembolic stroke, as well as its other determined and undetermined causes, 1549 patients with 754 pontine infarctions were included in the analysis. The prevalence of diabetes in the pontine infarction group was higher than that in the nonpontine infarction group (42.7% vs. 31.4%, *P* < 0.05). After adjusting for confounding factors, diabetes was an independent risk factor for pontine infarction (OR 1.63, 95% CI 1.27-2.09, *P* < 0.05). For small vessel occlusion, diabetes was also more common in the pontine infarction group (43.2% vs. 30.0%, *P* < 0.05). Multivariate analysis also showed that diabetes was an independent risk factor for pontine infarction (OR 1.80, 95% CI 1.32-2.46, *P* < 0.05).

**Conclusion:**

In comparison with the nonpontine infarction subtype of posterior circulation stroke, patients with pontine infarction had a higher prevalence of diabetes, and diabetes was an independent risk factor for pontine infarction.

## 1. Introduction

As the population ages, the prevalence of diabetes has increased dramatically in recent decades. It has increased from less than 1% to 11.6% in China, which has the largest diabetic population [1]. Diabetes is a fundamental issue for patients with atherosclerosis, and it has been recognized as a well-established risk factor for ischemic stroke [2]. Many studies have demonstrated that diabetes is significantly associated with stroke location [3, 4] because there is more diabetes in posterior circulation stroke than in anterior circulation stroke [3]. The possible mechanism was that the vertebrobasilar artery was more sensitive to hyperglycemia. Takahashi et al. found that diabetic patients had more plaques in the vertebrobasilar artery. Moreover, the vertebrobasilar artery plaques in diabetic patients were prone to rupture [5]. Thus, more attention should be given to diabetes in posterior circulation stroke.

Within the posterior circulation, the anatomy and physiology of the artery were heterogeneous among the different segments. Thus, the prevalence of diabetes may vary among the different subtypes of posterior circulation stroke. Pontine infarction is the most common subtype and accounts for more than 40% of posterior circulation strokes [6]. The prevalence of diabetes in pontine infarction varies from 28.5% to 59.4% [6, 7]. In comparison to nonpontine infarction, diabetes was more common in pontine infarction [8]. Whether the posterior circulation or diabetes has a unique role in pontine infarction remains unclear. Therefore, it is necessary to compare the prevalence of diabetes in pontine infarction with its prevalence in other subtypes of posterior circulation stroke, a comparison that has been lacking in previous reports.

In the present study, we aimed to investigate the prevalence of diabetes in pontine infarction and to compare it with that in nonpontine subtypes of posterior circulation stroke.

## 2. Methods

### 2.1. Study Design and Setting

This was a multicenter case-control study, which was conducted from November 1, 2018, to February 28, 2021. The study was approved by the Ethics Committee of the Second Affiliated Hospital of Guangzhou Medical University and Longhua Hospital. Informed consent was signed by the patient or the patient's authorized person.

### 2.2. Participants

Patients at the Second Affiliated Hospital of Guangzhou Medical University and Longhua Hospital were consecutively screened and enrolled if they met the following inclusion criteria: (1) older than 18 years old; (2) symptoms of acute stroke; (3) new infarction identified by diffusion-weighted imaging (DWI) within 7 days of stroke onset; (4) lesion located in the territory of posterior circulation; (5) intracranial and extracranial cerebral vessels evaluated by ultrasound, CTA, MRA, or DSA; and (6) infarction caused by atherosclerosis and arteriosclerosis. Patients were excluded if they met any of the following criteria: (1) missing clinical or imaging information; (2) nonatherosclerotic stroke; (3) brain tumor; (4) intracranial metastatic tumor; (5) intracerebral hemorrhage; or (6) traumatic brain injury.

### 2.3. Variables

A current smoker is defined as someone who smokes on some days or every day. An ex-smoker is defined as someone who has smoked ≥100 cigarettes in their lifetime but has not smoked in the past 28 days [9].

A former drinker is defined as someone who once drank but who did not drink during the past year; a current light drinker is defined as someone who in the past year has had 1-12 drinks but has 3 drinks or fewer per week on average; and a current heavy drinker is defined as someone who has had at least 3 drinks per week on average over the past year [10].

Hypertension is diagnosed when someone's systolic blood pressure in the office or clinic is ≥140 mmHg and/or their diastolic blood pressure is ≥90 mmHg following repeated examinations [11].

Diabetes is diagnosed when someone has the classical symptoms of hyperglycemia or hyperglycemic crisis with HbA1c ≥ 6.5% (the test should be performed in a laboratory using a method that is NGSP certified and standardized to the DCCT assay), fasting plasma glucose ≥ 7.0 mmol/L (fasting is defined as no caloric intake for at least 8 h), 2 h plasma glucose ≥ 111 mmol/L during a 75 g oral glucose tolerance test (OGTT), or a random plasma glucose ≥ 11.1 mmol/L. Poorly controlled diabetes was defined as HbA1c ≥ 7%. Someone with an HbA1c level of less than 7% is considered well controlled [12].

The classification of stroke subtypes using Trials of Org 10172 in Acute Stroke Treatment (TOAST) is as follows: (1) large-artery atherosclerosis, (2) cardioembolism, (3) small-vessel occlusion (SVO), (4) stroke of other determined etiology, and (5) stroke of undetermined etiology [13]. Stroke caused by large artery atherosclerosis and SVO were included in the analysis.

Information about medication usage was collected before stroke onset.

Pontine infarction was defined as an acute infarction located in the pons regardless of whether there were infarctions in other areas. Nonpontine infarction was defined as an acute infarction located in the extrapontine area.

### 2.4. Statistics

Differences in the continuous variables were compared using the test of homogeneity of variance. Student's *t-*test was used when the normality assumption was met; otherwise, the equivalent nonparametric test was used. Differences in the categorical variables were compared using Pearson's chi-square test with post hoc analysis. Missing data points and risk factor variables recorded as unknown did not exceed 3% for any single variable. A univariate binary logistic regression analysis was performed to determine the effects of the independent variables. Individual variables with a *P* value < 0.1 in the univariate analysis were used in the multivariable regression analysis, and the results were expressed as odds ratios (ORs) and 95% confidence intervals (CIs).

## 3. Results

### 3.1. Participants

**Figure 1:**
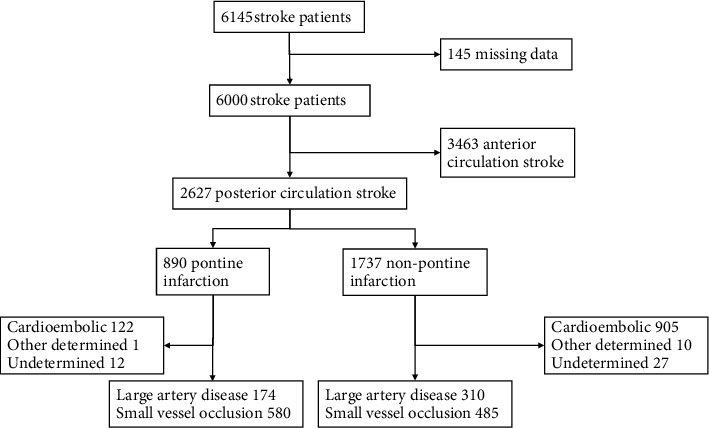
Flow diagram.

As shown in Figure 1, a total of 6145 stroke patients were screened. Posterior circulation strokes were found in 2627 patients with a total of 890 pontine infarctions. SVO was the leading cause of pontine infarction (65.2%, 580/890), while cardioembolism was the leading cause of nonpontine infarction (52.1%, 905/1737). Considering the limited effect of diabetes on cardioembolic stroke, as well as its other determined and undetermined causes, these cases were excluded from the analysis. Thus, 1549 subjects with 754 pontine infarctions and 795 nonpontine infarctions were included.

### 3.2. Association of Diabetes with Pontine Infarction

For the patients with pontine infarction, 42.7% (322/754) had diabetes, which was higher than the percentage of diabetic patients with nonpontine infarction (31.4%, 250/795). Most of the patients with diabetes were diagnosed previously. The baseline characteristics are shown in Table 1. Univariate analysis showed that hypertension, TC, LDL, diabetes, and fasting plasma glucose were significantly different between pontine and nonpontine infarction patients (Table 2, *P* < 0.05). Multivariate analysis showed that diabetes was an independent risk factor for pontine infarction (OR 1.63, 95% CI 1.27-2.09, *P* < 0.05, Table 2).

### 3.3. Association of Diabetes with SVO-Induced Pontine Infarction

A total of 580 patients had pontine infarction caused by SVO, and there was a higher prevalence of diabetes in these patients than in patients with nonpontine infarction (43.2% vs. 30.0%, *P* < 0.05, Table 3). Univariate analysis showed that age, hypertension, LDL, and diabetes were significantly different between pontine and nonpontine infarction patients (Table 4). Multivariate analysis showed that diabetes was an independent risk factor for pontine infarction in SVO (OR 1.80, 95% CI 1.32-2.46, *P* < 0.05, Table 4).

## 4. Discussion

In the present study, 42.7% of patients with pontine infarction had diabetes, which was higher than that of patients with nonpontine posterior circulation stroke. Diabetes was an independent risk factor for pontine infarction even after adjusting for confounding factors. Our study demonstrated the unique role of diabetes in pontine infarction. More attention should be given to diabetes in pontine infarction.

Our data showed that the prevalence of diabetes in pontine infarction patients was 42.7%. The patients in our study were Chinese. The diabetes prevalence in pontine infarction varied from 28.5% to 59.4% in previous studies, which recruited both Asians and non-Asians (Table 5). The mean prevalence of diabetes derived from the historical data was 39.0%, which was very close to our prevalence (Table 5). Thus, the prevalence of diabetes in pontine infarction was not affected by ethnic origins. This prevalence was much higher than that of nonpontine stroke. The prevalence of diabetes in the HERMES meta-analysis was 16.2% [14]. In the ECASS III trial, 15.7% of subjects had diabetes [15]. In the CHANCE trial, which mainly included Chinese stroke patients, 21.1% had diabetes [16]. In a head-to-head study, Nakase et al. found that the rate of diabetes was higher in patients with pontine infarction than in those without pontine infarction. However, nonpontine infarction includes both anterior and posterior circulation strokes [17]. Considering the higher rate of diabetes in posterior circulation stroke [3], one cannot exclude the possibility that it was because pontine infarction is a type of posterior circulation stroke. In the present study, 31.4% of patients with nonpontine infarction had diabetes, which was less than that of pontine infarction patients. Even after adjusting for confounding factors, diabetes was an independent risk factor for pontine infarction.

We found that SVO was the most common mechanism of pontine infarction, which was consistent with other studies [6]. Diabetes was associated more with SVO. Li et al. found that patients with pontine infarction caused by SVO were more likely to have diabetes than those with SVO-induced nonpontine infarction in the anterior circulation [18]. Thus, we performed a further subgroup analysis of patients with SVO. Among the patients with SVO, diabetes was also more common in pontine infarction than in nonpontine infarction of posterior circulation stroke. All of this evidence suggested that diabetes has a unique role in pontine infarction.

The arteries supplying the pons are the pontine arteries, which come off at right angles from the basilar artery. The mechanism underlying the greater susceptibility of the basilar artery to diabetes remains unknown [19]. Calcification in atherosclerotic plaques may predispose them to rupture by inducing mechanical instability. Calcification of basilar artery plaques was significantly related to diabetes [20]. The degree of calcification was positively related to the level of blood glucose [21]. An experimental study showed that relaxation of the basilar artery was negatively affected by diabetes [22], which resulted in constriction of the basilar artery to promote atherosclerosis [23]. The mechanism might be that large-conductance Ca^2+^-activated potassium channels in basilar artery smooth muscle cells were impaired by diabetes [24].

Most of the patients with previously diagnosed diabetes received treatment, including drug and/or nondrug treatments. The rate of treatment was higher than the average in China. However, for the majority of patients receiving treatment, their diabetes was poorly controlled. Higher HbA1c was related to early neurologic deterioration and the long-term poor prognosis of pontine infarction [25, 26]. Moreover, HbA1c could promote the progression of TIA to stroke in the posterior circulation [27]. Therefore, a tight glucose control strategy was necessary for the prevention of pontine infarction.

There were some limitations of the study. First, the diagnosis of infarction in the present study was based on DWI images. However, some patients, especially those with brainstem strokes, had DWI-negative strokes. This would lead to some bias. Second, the diagnosis of SVO was mostly made based on the infarction distribution. Some studies have used high-resolution MRI (HR-MRI) to detect branch artery disease [28]. Some of our subjects also received HR-MRI. However, not all branch arteries could be detected by HR-MRI. Finally, although our study was one of the largest in terms of the number of pontine infarction patients, more subjects and more studies are needed to investigate the relationship between diabetes and pontine infarction.

## 5. Conclusion

In conclusion, the prevalence of diabetes was higher in pontine infarction than in the other subtypes of posterior circulation stroke, and diabetes was an independent risk factor for pontine infarction.

## Figures and Tables

**Table 1:** Pontine infarction vs. nonpontine infarction.

	Pontine infarction	Nonpontine infarction	*P*
*N*	754	795	
Male (%)	475 (63.0%)	527 (66.2%)	0.184
Age (y)	66.97 ± 11.95	66.03 ± 12.15	0.118
Smoking (%)	205 (27.2%)	238 (29.9%)	0.432
Ex-smoker (%)	47 (22.9%)	50 (21.0%)	
Current smoker (%)	158 (77.1%)	188 (79.0%)	
Drinking	82 (10.9%)	95 (11.9%)	0.154
Former drinker (%)	26 (31.7%)	15 (15.8%)	
Current heavy drinker (%)	52 (63.4%)	69 (72.6%)	
Current light drinker (%)	4 (4.9%)	11 (11.5%)	
Hypertension (%)	584 (77.4%)	582 (73.2%)	0.051
Hyperlipidemia			
TC (mM)	4.79 ± 1.26	4.61 ± 1.19	<0.01
LDL (mM)	3.18 ± 1.08	3.03 ± 1.00	<0.01
HDL (mM)	1.05 ± 0.53	1.04 ± 0.25	0.676
TG (mM)	1.81 ± 1.49	1.71 ± 1.14	0.142
Uric acid (*μ*M)	353.77 ± 111.25	361.10 ± 111.38	0.201
Previous stroke or TIA (%)	146 (19.4%)	176 (22.1%)	0.183
Medicine			
Antiplatelet (%)	205 (27.2%)	186 (23.4%)	0.080
Statin (%)	55 (7.3%)	70 (8.8%)	0.283
Antihypertensive agent (%)	335 (44.4%)	349 (43.9%)	0.178
Diabetes (%)	322 (42.7%)	250 (31.4%)	<0.01
New diagnosis (%)	55 (17.1%)	50 (20.0%)	
Previously diagnosed	267 (82.9%)	200 (80.0%)	
No treatment (%)	23 (8.6%)	18 (9.0%)	
Poorly controlled (%)	208 (77.9%)	136 (68.0%)	
Well-controlled (%)	37 (13.8%)	46 (23.0%)	
Fasting plasma glucose (mM)	6.46 ± 2.93	6.11 ± 2.60	0.022

TC: total cholesterol; LDL: low-density lipoprotein; HDL: high-density lipoprotein; TG: triglyceride; TIA: transient ischemic attack.

**Table 2:** Multivariate logistic regression of pontine infarction.

	Univariate		*P*	Multivariate		*P*
	OR	95% CI		OR	95% CI	
Male	0.87	0.70-1.07	0.176			
Age	1.00	0.99-1.02	0.121			
Smoking	0.92	0.82-1.04	0.205			
Drinking	0.89	0.75-1.04	0.146			
Hypertension	1.26	0.99-1.59	0.051	1.13	0.86-1.48	0.401
Hyperlipidemia						
TC	1.12	1.04-1.12	0.009	1.01	0.83-1.23	0.887
LDL	1.16	1.05-1.27	<0.01	1.15	0.91-1.44	0.242
HDL	1.05	0.82-1.35	0.678			
TG	1.06	0.98-1.15	0.142			
Uric acid	1.00	0.99-1.00	0.201			
Previous stroke or TIA	0.84	0.66-1.08	0.184			
Medicine						
Antiplatelet	1.23	0.98-1.55	0.078	1.21	0.96-1.54	0.110
Statin	0.82	0.56-1.18	0.285			
Antihypertensive agent	1.14	0.99-1.31	0.074	1.06	0.90-1.26	0.487
Diabetes	1.61	1.31-1.98	<0.01	1.63	1.27-2.09	<0.01
Fasting plasma glucose	1.04	1.01-1.09	0.018	0.99	0.95-1.04	0.734

TC: total cholesterol; LDL: low-density lipoprotein; HDL: high-density lipoprotein; TG: triglyceride; TIA: transient ischemic attack.

**Table 3:** Pontine infarction vs. nonpontine infarction with SVO.

	Pontine infarction	Nonpontine infarction	*P*
*N*	580	486	
Male (%)	370 (63.8%)	321 (66.0%)	0.442
Age (y)	67.14 ± 11.76	65.38 ± 11.94	0.018
Smoking (%)	158 (27.2%)	142 (29.2%)	0.774
Ex-smoker (%)	34 (21.5%)	30 (21.2%)	
Current smoker (%)	124 (78.5%)	112 (78.8%)	
Drinking	61 (10.5%)	56 (11.5%)	0.123
Former drinker (%)	18 (29.5%)	9 (16.1%)	
Current heavy drinker (%)	41 (67.2%)	40 (71.4%)	
Current light drinker (%)	2 (3.3%)	7 (12.5%)	
Hypertension (%)	444 (76.6%)	343 (70.6%)	0.028
Hyperlipidemia			
TC (mM)	4.81 ± 1.29	4.66 ± 1.20	0.062
LDL (mM)	3.20 ± 1.10	3.04 ± 1.00	0.018
HDL (mM)	1.04 ± 0.28	1.04 ± 0.25	0.990
TG (mM)	1.85 ± 1.61	1.82 ± 1.28	0.791
Uric acid (*μ*M)	356.08 ± 107.43	356.89 ± 103.66	0.868
Previous stroke or TIA (%)	108 (18.6%)	102 (20.9%)	0.330
Coronal heart disease (%)	44 (7.6%)	37 (7.6%)	0.996
Medicine			
Antiplatelet (%)	157 (27.0%)	116 (23.9%)	0.228
Statin (%)	40 (6.9%)	46 (9.5%)	0.131
Antihypertensive agent (%)	247 (42.6%)	207 (42.5%)	0.232
Diabetes (%)	251 (43.2%)	146 (30.0%)	<0.01
New diagnosis (%)	38 (15.1%)	31 (21.2%)	0.374
No drugs (%)	15 (5.9%)	9 (6.1%)	
Poorly controlled (%)	161 (64.1%)	79 (52.0%)	
Well-controlled (%)	33 (13.1%)	27 (18.5%)	
Fasting plasma glucose (mM)	6.45 ± 3.04	6.12 ± 2.62	0.058

TC: total cholesterol; LDL: low-density lipoprotein; HDL: high-density lipoprotein; TG: triglyceride; TIA: transient ischemic attack.

**Table 4:** Multivariate logistic regression of pontine infarction with SVO.

	Univariate		*P*	Multivariate		*P*
	OR	95% CI		OR	95% CI	
Male	0.91	0.70-1.17	0.441			
Age	1.01	1.00-1.02	0.019	1.01	1.00-1.02	0.052
Smoking	0.95	0.82-1.07	0.483			
Drinking	0.89	0.73-1.08	0.240			
Hypertension	1.36	1.03-1.79	0.032	1.24	0.94-1.65	0.132
Hyperlipidemia						
TC	1.10	0.99-1.22	0.058	0.93	0.74-1.16	0.521
LDL	1.15	1.02-1.30	0.021	1.26	0.97-1.65	0.089
HDL	1.00	0.63-1.58	0.987			
TG	1.01	0.93-1.10	0.789			
Uric acid	1.00	0.99-1.00	0.362			
Previous stroke or TIA	0.86	0.64-1.16	0.332			
Medicine						
Antiplatelet	1.18	0.90-1.56	0.230			
Statin	0.71	1.45-1.10	0.131			
Antihypertensive agent	1.15	0.97-1.36	0.108			
Diabetes	1.73	0.33-2.23	<0.01	1.80	1.32-2.46	<0.01
Fasting plasma glucose	1.04	0.99-1.09	0.062	0.99	0.94-1.04	0.601

TC: total cholesterol; LDL: low-density lipoprotein; HDL: high-density lipoprotein; TG: triglyceride; TIA: transient ischemic attack.

**Table 5:** Previous reports of diabetes in pontine infarction.

Year	Author	Diabetes	Pontine infarction	Prevalence (%)
1994	Toyoda [29]	29	73	39.73
2002	Kumral [30]	45	150	30.00
2005	Vemmos [31]	29	100	29.00
2008	Liang [32]	4	14	28.57
2009	Kwon [33]	49	96	51.04
2009	Liang [34]	4	17	23.53
2010	Aoki [35]	25	51	49.02
2010	Klein [36]	13	45	28.89
2012	Oh [37]	87	200	43.50
2013	Feng [38]	25	81	30.86
2013	Ju [39]	60	101	59.40
2014	Nakase [40]	13	38	34.20
2015	Feng [41]	19	55	34.54
2015	Lim [42]	36	87	41.38
2016	Huang [43]	147	265	55.47
2016	Wilson [7]	189	619	30.53
2017	Gokcal [44]	69	120	57.50
2017	Lapa [45]	31	59	52.54
2018	Zhou [6]	61	175	34.86
2019	Huang [46]	372	1003	37.09
Total		1307	3349	39.03

## Data Availability

All data was available.
